# Views on clinical trial recruitment, biospecimen collection, and cancer research: population science from landscapes of the Haudenosaunee (People of the Longhouse)

**DOI:** 10.1007/s13187-016-1067-5

**Published:** 2016-07-09

**Authors:** Rodney C. Haring, Whitney Ann Henry, Maui Hudson, Elisa M. Rodriguez, Maile Taualii

**Affiliations:** 1 Roswell Park Cancer Institute, Office of Cancer Health Disparities, Cancer Prevention and Population Sciences, Elm & Carlton Streets, Buffalo, NY 14263 USA; 2 Environmental Research Institute—Faculty of Science, Dept of Management Communications—Waikato Management School, University of Waikato: Maori & Indigenous Governance Centre—Faculty of Law, Private Bag 3105, Hamilton, 3240 New Zealand; 3 Office of Public Health Studies, Native Hawaiian and Indigenous Health, 1960 East-West Road, Honolulu, HI 96822 USA

**Keywords:** Bio-specimen research, Bio-banking, Ethics, Native American, American Indian, Haudenosaunee, Iroquois, Cancer, Cancer education, Cancer prevention, Clinical trial, Recruitment, Minority, Patient communication

## Abstract

Biomedical research in culturally distinct communities is often a challenge. Potential barriers to participation occur because science is presented in a format that lacks cultural acknowledgement. Investigations may also fail to showcase beneficial relevance to the communities or include them in true partnership. The history of biomedical research within Native American societies has been complicated by these issues. Historical trauma among many Native groups sometimes transcends into contemporary challenges in both recruitment to and participation particularly in biobanking research. The participants for this study included members of the Haudenosaunee, the People of the Longhouse. Native Americans, including the Haudenosaunee, endure some of the worst health disparities in the country. These include high rates of cancer, obesity, and diabetes which may be linked at least partially to genetic predisposition. Results from a Haudenosaunee urban population shared response on ways to improve recruitment strategies for biospecimen, cancer, and other health-related clinical trials. Mixed methods approaches were used, and community responses indicated the importance of creating trust through respectful partnership; promoting culturally appropriate recruitment materials; the need for a greater understanding of consenting and signature processes; the necessity for concise summary sheets; and a desire to have information that community member understand. Discussion items also include international Indigenous perspectives to biobanking and genetic-related health disparity research.

## Introduction

Research in minority, underserved, ethnically diverse, or culturally distinct communities is often a challenge. Whether it is a small survey, an interview or, on the other end of the spectrum, biospecimen collection, the challenges are real and can be seen in a multitude of ways. Potential challenges include lack of participation because the research seems complicated or may not have been presented in a language or format that is culturally acceptable to the community. Investigations may also fail to showcase beneficial relevance to the communities with whom the project intends to partner. Whatever the cause of hesitation, it is an important topic, a topic that has direct relevance to minority populations, their people, and their future.

Although these issues are not totally missing in published scientific literature, they are not presented often enough to be visible in many research institutions or within community settings. This article highlights this subject matter and provides recommendations. The focus of this paper is to provide current results from a Native American urban population focused on the voices, insights, experiences, and perceptions from the community on ways to improve recruitment strategies for cancer and other health-related clinical trials. These participants included members of the Haudenosaunee, the People of the Longhouse. The Haudenosaunee are a Confederacy of Tribes in the Northeastern areas of New York State whose bloodlines are distinctly related through clan systems, language, and traditional practices. The Haudenosaunee Confederacy includes the six nations: Mohawk, Oneida, Onondaga, Cayuga, Seneca, and Tuscarora.

Overall, responses from the Haudenosaunee provide helpful steps for respectful partnership-building and for understanding how recruitment in special populations is translated. The article will also educate on the philosophies of community partnerships that honor and have the ability to shape meaningful research agendas to move the science forward from researchers, to communities, and for future generations.

## Background and Significance

The history of research within Native American communities throughout the USA has not left the best impression. Rather, it has often led to mistrust of the government and researchers associated with the government. Many community members to this day are still skeptical of research and do not want to be seen as “guinea pigs” [22]. Historical mistrust also traces back just a few generations, deriving from “gift blankets” infected with smallpox [25]. This story of germ warfare, ironically, has ties to the Haudenosaunee lands now named Amherst, N.Y. This area was given to Sir Jeffery Amherst by King George III for his military services. According to historical narratives, shared in the Journal of William Trent, under Amherst’s watch, “we gave them two blankets and an handkerchief out of the Small Pox Hospital. I hope it will have the desired effect” [16]. This deep historical trauma among many Native American communities often transcends into contemporary challenges in both this population’s recruitment to and participation in clinical trials as well as biobanking research.

Native Americans endure some of the worst health disparities and inequalities in the USA, of which many are preventable diseases. These include high rates of diabetes, alcohol-related deaths, injuries, suicide, and lower rates of cancer screening ([1, 13] (a); [14] (b); [15, 34]). These disparities may be linked to genetic predisposition, socioeconomic status, access to and utilization of services, and cultural factors [33]. These are also likely tied to historical trauma and long-term stress related to forced attendance at boarding schools and even modern-day stressors related to issues of team mascots, as noted by Taylor [30]): “The personal nature of the exploitation of Native people and their societies and cultural displays is an element that serves to connect…the issue and self-worth of Native American youth.” Thus, it does not come as a surprise to see little participation from Native Americans in research today—especially related to clinical trials and biobanking.

In order to gain the confidence of Native communities, it is important for research team members to be aware of the entire process. Hiratsuka, Brown, Hoeft, and Dillard [10] explained that “A clear and extensive process of informed consent and continued improvements in sharing results may enhance the transparency of research intent, conduct, and use of obtained results among Alaska Native people.” It is important to Native people to know what their blood or tissue is used for and how the clinical trial is going to help their community. It is also important for these communities to be aware of historical occurrences related to biospecimen research and what to look for. This sharing may include monumental cases including the occurrence at Havasupai Nation in which Arizona State University researchers “violated consent forms” and used their blood samples for something completely different (Potkonjak (2004) in [32]).

Fortunately, relationships are improving between researchers and Native American communities. Following the Indian Self-Determination and Education Assistance Act of 1975, Alaska Native Tribal Health Organizations have assumed management of their healthcare system and, in 2004, they assumed shared ownership of the Alaska Area Specimen Bank (AASB), a collection of stored samples dating back to the 1940s from U.S.-funded Alaska research projects, that resides in the Alaska Area Specimen Bank in Anchorage, AK [10]. With these new processes of trust and collaboration emerging in the Arctic region, the AASB plays an important role in shaping biospecimen management, control, and utilization. As such, the ASSB may be in line to inform and extend federal regulation beyond the individual—towards biospecimen regulations aimed for the collective—including the safety of culture, tradition, and religion (Gachupin & Freeman in Solomon et al., [25]).

Overall, communities must remain a part of the process and discussion of ownership of the specimens is likely a good start—by doing so, it allows the community to see and have access to their specimens at all times. Along with that, Hiratsuka, Brown, Hoeft, and Dillard [10] also mentioned that information germane to the motivation and intent of researchers including specifics of specimen storage and destruction was important knowledge to tribes. This is crucial when dealing with Native communities; it is important to give tribes the choice of what to do with a specimen after researchers are finished using it. This may include providing spiritual ceremonies honoring those specimen donors.

## Methods

The aim of the research project was to ask participants from various areas and populations from across the USA and border regions of Canada about their perceptions of, comments on, and suggestions for a series of educational flash cards designed to explain clinical research, recruitment, and biospecimen-related investigation, with a focus on health disparity-related inquiry in the cancer research realm. These cards were described in the previous literature [31]. Participants ranged from Native American populations and African American communities to Caucasians in rural and Appalachian regions. This paper focused on Native American responses only.

### Study Sample and Design

The first phase of the project was based on visual review of the cards with qualitative feedback. The second phase of the project focused on unique responses to redesigned cards based on phase 1 results. The objective of these cards in both phases 1 and 2 was to help improve the communication between potential research participants and clinical trial recruiters. Descriptive statistics from phase 2 were used to support qualitative findings.

Participants were self-identified members of Native American tribes or bands from urban populations in the Northeastern area of the USA and Southern Ontario, Canada. All participants were from tribes or bands of the Haudenosaunee (Iroquois or People of the Longhouse), people of other tribal affiliations living with or among the Haudenosaunee, or residents in off-territory areas situated in their aboriginal homelands of Western New York or Southern Ontario, Canada. All interviews were conducted in the USA. In total, 16 Native community members were recruited for interviews and qualitative analysis in this first phase. All participants had at least one family member affected by cancer and four of the Native American participants noted experience with research recruitment. Of the 16, three were female cancer survivors, nine were non-cancer-community members, and four participants withdrew due to a delay in the project initiation. Phase 2 included 20 in-person interviews, which were followed immediately by in-person data collection via survey. These participants were different from phase 1 but were from the same geographic region. In total, 35 Native Americans were recruited with 28 providing feedback.

Stories and responses were collected using questionnaires, and forms approved by Northwestern University’s IRB and subsequently reviewed and approved through a Memorandum of Understanding with leaders in the Native American area selected as the community-based partner. Phase 2 was approved by Roswell Park Cancer Institute’s IRB. Researchers conducted interviews and took notes throughout the interview process. These notes formed the basis for qualitative analysis.

### Analysis: Qualitative

Analysis was completed using the process of coding, categorizing, and the linking categories method outlined by Glaser [8] and Strauss and Corbin [24]. Through this methodology, results of the analysis yielded a beginning/summary awareness and process for recruitment and retention methods specifically within the Haudenosaunee landscape. Ultimately, the process aimed to keep in mind the research goals of addressing gaps in recruitment strategies, helping inform—retention strategies for clinical trial studies within minority populations, and beginning to build a template that may be useful for study and collaborative processes with Native Americans and other minority communities.

The analysis component of the project was exploratory in nature and integrated features of grounded theory for review. Grounded theory used in previous minority biospecimen research analysis [11] helped construct a framework of categorical processes that explained the relationships between how the project was meaningful at the micro, mezzo, and macro levels of utilization as it pertained to Native American communities. Categories and their intertwining properties and dimensions that emerged from the data assisted in the explanation of how minority participants, through the lens and awareness of their community, brought meaning to their voices.

Further, the model, in the form of a recruitment flash card and informational gathering session, helped increase the project team’s awareness and assist in explaining the process of collaboration, gaps of knowledge, and the need for sharing the voices for how to successfully implement health research projects in minority populations—specifically Native Americans.

### Qualitative Results

#### Creating Trust through Respectful Partnerships (core-category)

The core-category in which all the other categories interact is the theme related to *trust*. From an administrative standpoint, it is important for research teams to be fully prepared to be part of the community-based participatory research process. This includes understanding the administrative mechanism of providing contractual information to the tribal government or community center board when asked, to be available for questions, and to be responsive to time-frames. By doing so, the communication and responsiveness begins to build relationships for proposed research but also sets in motion a foundation for future collaboration. Further communicating with the partnering entities regarding research becomes a means of acknowledging sovereignty or Native urban center placement within the geographic region. Accomplishing this helps maintain partnerships and relationships (Randall in Solomon et al., [25]). Conversely, if these relationships are not developed or in-place it may be difficult for the community to support next-phase projects.

This process was also noted among Kanaka Maoli, Native Hawaiians, and their views on biobanking. Native Hawaiian understandings and stances were explored through in-depth focus groups by Taualii et al.[26]). Findings highlighted the need to build trust and mutual understanding between researchers and Indigenous communities. The acronym “G.R.E.A.T. Research” was created, which outlined the key areas identified as necessary for quality research that delivers value to Hawaiian communities, involve the target populations in *Governance*, provide participants with the option to *Re*-*consent* each tissue use, ensure *Education* will be ongoing and informative, include *Accountable* researchers from the population of focus, maintain an open and *Transparent* process, and ensure that the *Research* reflects the priorities of the participant communities. Native Hawaiian participants informed Taualii et al. [26] that if the outlined guidelines were followed it could help to build trust and increase participation.

Revisiting the phase 1 Haudenosaunee voices, trust-building, and relationship building was important. This echoed the voices of the Alaska Natives, who shared that a lot of mistrust can come from “lack of awareness of results dissemination of previous studies” [10]. Petereit and Burhansstipanov [22] also noted the importance of this by indicating that it can take years of going in and building trust in Native communities before researchers can even apply for support.

In summary, Native and Indigenous communities world-wide have to be cognizant and informed of research processes occurring in their sovereign or urban landscapes. Research teams wishing to conduct research should also consider ongoing and in-depth involvement with the tribe or urban center, the hiring and training of local community members, and the consideration of gender roles in Native societies—specifically that of the women, who often assist the men—and are often charged with maintaining the mechanisms for the continuation of traditional cultures in the modern age [25]. Without the trust of the community, researchers may not have the opportunity to continue their work.

#### Culturally-Appropriate Recruitment Materials (category)

Participants in phase 1 indicated that flash cards were not visually appealing or marketable to Native American communities. This was represented by a community member, who said, “It appeared that this study was not for me or us (Native Americans). There was little imagery that would show that Natives were to be part of the project.” This phenomenon has also been noted in other clinical trial recruitment study within economically disadvantaged societies [19]. Haudenosaunee respondents in our sample shared a need for culturally appealing recruitment materials and imagery, including tribal languages. Guadagnelo et al. [9] pointed out that “patient education materials were translated into the Lakota language,” which allows Native American patients to understand the material. Thus, it would likely have the same effect on recruitment materials. It is a key element for potential research participants to be able to relate, feel included, and understand the recruitment material in order for them to continue to participate in future research endeavors.

#### Understanding the Signature Process (Category)

Another important area shared by a number of phase 1 participants is the signature process. Participants felt this needed a little more emphasis and explanation. Participants shared that potential study enrollees should understand exactly what they are signing. Another indicated that the signing process should be done carefully and in more detail. As one participant stated, “Know what you are signing up for.” Concerns about the lack of detailed information on trials were also evident in previous literature [20]. Further, the results found among the Haudenosaunee are reflective of Alaska Native responses to research involving human specimens, for which people desired extensive disclosure of information beyond that typically provided in consent and results-dissemination processes [10]. It should be noted, however, that in phase 2 of this project, after cards were revised, 95 % of the participants agreed that when medical words were used they were explained. Further, 95 % of phase 2 participants also believed that it was true that they were able to refuse signature on the consent if they did not want to participate.

Respondents also shared that community language, familiar use of jargon, or more specifically, the breakdown of medical terminology into more lay language were important. Thus, a property of incorporating lay language was seen in the suggested process of understanding the signature process. One respondent indicated that some, not all, of the cards could be improved by including language that non-health practitioners and the general public would understand. By not doing so, people in the Native community may not participate. Another stated that some cards would benefit from the use of common language. For instance, using the phrase, “Your name will not be attached to the information collected,” rather than the word “de-identified.”

#### Providing a Summary Sheet (category)

Participants also shared that having a “key point” or “summary sheet” that shares important features of the study would be very helpful. One participant shared an information sheet that was given to her when she donated biospecimens for cancer research. She recommended that it could have been simplified and, if there were summary cards or accompanying summary sheets that were key take-away points, it would have been easier for her to understand. This was also reflected by others interviewed. Thus, the use of culturally acceptable flash cards with summary points seems feasible.

## Summary

In conclusion, tribal members were very cautious about how research was conducted in their communities. Just over 25 % of phase 2 participants indicated they would be very likely to donate a biospecimen, while 46 % said they would be somewhat likely to donate, and 27 % were neutral about donating. Many phase 1 respondents were guarded about the overall process of biobanking and genetic related investigations. Here, deeper explanation prior to a signature may be helpful. That being said, two cancer patients interviewed in phase 1 previously provided biospecimen samples for research purposes. They both felt it was their duty to participate in research to help future generations. One, however, questioned, “How many studies were going to be conducted from my donation?” She also indicated that the flash cards were more informative than the one-page sheet given prior to her operation and subsequent biological sample donation. Participants further noted that recruitment strategies needed to be in community language with community appeal. Of great importance was the emphasis on “trust” prior to in-depth study. Building a rapport between the researchers and the governing body of the Nation or board is essential. Lastly, recruitment strategies must include lay language, information about the length of the study, commitment, and the rationale of the study.

## Discussion

Perhaps the reasons for the slow translation of Northeastern U.S. health research procedures include a number of items. One may be the lower number of federally recognized Indian tribes in the Northeast compared to other areas of the USA that have had a longer history of research relationships with universities and institutions. It may also be the case that universities and institutions of the Northeast are becoming more aware of the process of working in special population communities such as sovereign tribal lands. Whatever the case may be, it is crucially important that institutions of research be open-minded to community-based participatory research, community-driven processes that highlight beneficence, and transparency.

Hiratsuka et al. [10] made a comment on how communities want to be able to tell the difference between beneficence and personal gain on the researchers’ end. Another important aspect is having a lead researcher of Native descent. LaVallie et al. [18] mentioned that this was a major factor among Alaska Native communities and, by doing so, they felt a sense of cultural awareness that gained the trust of the community. Another question among Native communities is what are the “next steps” in the research process and beneficence: What will continue to happen to benefit the community with the findings? Hiratsuka et al. [10] mentions that these next steps are things the community needs to know. Much like the Alaska Native communities, other Native American communities plan ahead for the health and well-being of their future generations. This is exemplified by a passage shared by a Haudenosaunee leader (TedX Buffalo, https://www.youtube.com/watch?v=YyuSc_jkG-s, retrieved January 8, 2016):

Every action we take we have to be mindful seven generations up…that every action and decision we make…it has to ensure their well-being….just as we look back those seven generations…we give thanks to those that came before us….and that intergenerational thinking also plays part of the Good Mind….because it’s not just about the now but it’s about the legacy left us and that what we have to leave for our future generations—Michael Martin [36].

If tribes, Nations, and Native urban populations are going to participate in bio-medical research (i.e., biobanking), consideration of a plan to continue to help the community should be in place to share findings, intertwine Western models of science with Native theoretical concepts, and promote the generalizability of that which may have benefit for future generations. Further, federally recognized Indian Nations have the sovereign right through the Indian Self Determination Act to work collaboratively with biobanking centers to have input on the use of DNA from their families and ancestors. Though collaborative relationships, it may in fact be a way in which tribes and research institutions can jointly unlock codes to fight against chronic diseases, cancer, and other ailments that affect Native American populations disproportionately.

### Minority Community Context

The issues and concerns highlighted above by Native American communities with respect to biospecimen research practices have relevance to other diverse vulnerable and underserved populations. The concepts of community benefit, relevance to the community, and transparency as related to biospecimen donation for cancer research have been cited as issues to consider by both African American and Hispanic community participants in formative research on awareness and interest to participate in biobanking studies [7, 17, 23].

Additionally, Dang et al. [2] provided a cross-cultural comparison of three distinct community sites across the USA that collaborated with their local communities on the topic of biospecimen donation participation for cancer research. The sites partnered with participants from African American, three Asian American ethnic groups, as well as diverse Hispanics, and Whites. These centers in collaboration with their respective communities examined cognitive, communication, and sociocultural factors affecting biospecimen donation participation. Impediments to community participation in biospecimen donation for cancer research varied by the population(s) studied and included factors concerning cultural beliefs regarding blood or tissue to be donated; some community members described the feeling that research uses them as test monkeys; and others expressed a fear of blood and needles as a deterrent for donating [2].

There are definite areas of overlap in terms of concerns and topics addressed by the studies that included African American, Hispanic, and various Asian American participants with regard to their respective levels of awareness and interest in participating in biospecimen donation for cancer research and the concerns mentioned by Native American community members. Meade et al. [21] and colleagues described the development of their community-based tools to increase community awareness as it relates to biospecimen donation and the importance of inclusion and representation in this body of research from diverse communities. Both the lessons learned and the tools that have been collaboratively created through these formative pilot studies with diverse communities may provide useful templates for how to engage and partner with Native American communities on biospecimen donation for cancer research.

### International Perspectives

Similar perspectives are also emerging from Indigenous Maori communities in New Zealand, where a number of authors have proposed ways to enhance community control when Māori are involved in research. This includes Māori involvement at all levels of the research, Māori setting the research agenda, Māori determining the interpretation and dissemination of research outputs, and Māori involvement in governance of samples and data [4–6, 27–29, 35]. Tribal communities in New Zealand have stressed the importance of their involvement in governance of research and biobanking activities due to the level of risk for participants, the cultural importance of tissue, DNA, data, and the need for collaborative relationships to ensure benefits are realized in their communities [12].

Community involvement in the governance of research and biobanking activities has also been identified in the context of genome studies with Indigenous Australians [3]. Key points acknowledged by their Aboriginal Governance Committee included the need for a specific conceptual framework for genetic research and bio-banking in Aboriginal and Torres Strait Islander communities, continuing governance of samples and data, the development of principles for future data use, and engagement with community elders to ensure the cultural backbone for the project remains strong. The research team also engaged directly with communities through culturally specific focus groups, which identified ways in which genetics could be easily communicated through consent processes.

## Suggestions and Conclusion

These suggestions provide the framework for not only institutions and scientists, but also for tribes and urban centers that have the ability to coordinate with their respective Native Nations. Perhaps the most alarming finding is the lack of translational movement across minority and underrepresented populations in the past clinical and biospecimen research literature. Thus, suggested strategies include the progression of Native governments to use sovereign abilities to mobilize the science of biospecimen collection, storage, and direction of use. Doing this results in putting the action and planning into the control, oversight, and development of tribal governments, their officials, and tribal councils. Further, it allows Native Nations to create Indigenous ways of recruitment and to have the ability to secure a layer of trust that may not be available when outside institutions maintain authority over sample and research processes.

Globally, biospecimen research and clinical trials are important for the health of future generations and our co-existence with the environment in which we situate. It is crucially important that unique societies, under the right circumstances, contribute to the science—an emerging science that should encourage the respect of all societies, a science that is culturally appropriate and explains the “fine print” in the language and understanding of the people and populations with whom the study aims to partner and collaborate. Further, it is important that the science honor the sovereignty of Native Nations to contribute to the benefit of all generations. By doing so, the process advances the science of wellness and has the ability to bring full circle reductions and perhaps the elimination of health disparities for a wide variety of populations world-wide.
